# Radioiodination and Purification of [^131^I]β-CIT and [^131^I]FP-CIT with an Automated Radiosynthesizer

**DOI:** 10.3390/ph15010096

**Published:** 2022-01-14

**Authors:** Elisabeth Plhak, Edith Gößnitzer, Reingard M. Aigner, Herbert Kvaternik

**Affiliations:** 1 Department of Radiology, Division of Nuclear Medicine, Medical University of Graz, Auenbruggerplatz 9, A-8036 Graz, Austria; reingard.aigner@medunigraz.at (R.M.A.); herbert.kvaternik@medunigraz.at (H.K.); 2 Department of Pharmaceutical Chemistry, Institute of Pharmaceutical Sciences, University of Graz, Schubertstraße 1/I, A-8010 Graz, Austria; edith.goessnitzer@uni-graz.at

**Keywords:** iodine-131, β-CIT, FP-CIT, automated synthesis, radioiodo-destannylation

## Abstract

Dopaminergic transporter (DAT) imaging with single photon emission computed tomography (SPECT) is used to diagnose Parkinson’s disease and to differentiate it from other neurodegenerative disorders without presynaptic dopaminergic dysfunction. The radioiodinated tropane alkaloids [^123^I]FP-CIT and [^123^I]β-CIT enable the evaluation of the integrity of DATs. Commonly, the labeling of these compounds is performed by electrophilic substitution of the alkylstannylated precursors with radioactive iodine and following purification by HPLC or solid phase extraction (SPE). This work presents the first radioiodination of β-CIT and FP-CIT with no carrier added [^131^I]NaI on a Scintomics GRP synthesis module. Free iodine-131 and impurities were removed by SPE over a C-18 Sep-Pak cartridge. We achieved a radiochemical yield of >75% and a radiochemical purity of >98% with both compounds. Our development of an automated synthesis on a commercially available synthesizer ensures robust and efficient labeling of [^131^I]FP-CIT and [^131^I]β-CIT starting with low concentrated radioiodine.

## 1. Introduction

Parkinson’s disease is a neurodegenerative disorder characterized by the loss of dopaminergic neurons in the substancia nigra pars compacta. This leads to a decrease in the density of presynaptic dopamine reuptake receptors.

DAT imaging with single photon emission computed tomography (SPECT) or positron emission tomography (PET) tracers supports the clinical diagnosis of Parkinson’s disease. In addition, it helps to discriminate from motor syndromes not caused by the loss of dopaminergic neurons, such as essential tremors, dopa-responsive dystonia, and drug-induced Parkinsonism [1,2].

Clinical applied DAT SPECT imaging agents are tropane derivatives, such as β-CIT (Iometopane, 2β-carboxymethoxy-3β-(4-iodophenyl)tropane) and FP-CIT (Ioflupane, N-3-fluoropropyl-2β-carbomethoxy-3β-(4-iodophenyl)nortropane), both labeled with iodine-123 and technetium-99m labeled TRODAT ([2[[2-[[[3-(4-chlorophenyl)-8-methyl-8-azabicyclo[3,2,1]-oct-2-yl]-methyl](2-mercaptoethyl)amino]ethyl]amino]ethanethiolato(3-)-N2,N2’,S2,S2]oxo-[1R-exo-exo)]) [3]. The technetium-99m labeled ligand is convenient to prepare on-site using kits. However, it shows a higher imaging background and lower specificity than the iodine-123-labeled dopamine transporter ligands [4,5,6]. [^123^I]FP-CIT binds selectively to DATs and shows an accumulation within 3–4 h post-injection, which enables the exam to be completed in one day. Furthermore, the fast washout reduces the radiation burden on the basal ganglia. [^123^I]β-CIT has a higher affinity to DATs and 5-HT receptors and shows lower unspecific uptake in the brain than [^123^I]FP-CIT. However, the disadvantage of [^123^I]β-CIT is the slow pharmacokinetics. The maximum striatal uptake is reached within 14–24 h post-injection and requires a two day imaging protocol [7,8,9].

Commonly, the radiolabeling of β-CIT and FP-CIT is carried out by the radioiodo-destannylation of trialkylstannylated precursors [10,11,12,13,14]. For the electrophilic substitution, the radioactive iodide (I^−^) must be oxidized to iodine (I_2_) in an acidic solution using different oxidizing agents: hydrogen peroxide (H_2_O_2_)/hydrochloric acid (HCl) [11], peracetic acid/acetic acid (HOAc) [12], chloramine-T/HCl [10,13,14] or Iodo-Gen^®^/phosphoric acid (H_3_PO_4_) [13]. The crude product is purified by preparative HPLC followed by the evaporation of the solvent under reduced pressure. Alternatively, this time-consuming step can be replaced by SPE on a C-18 Sep-Pak cartridge [12,13].

This work presents the successful development of a suitable automated labeling route for radioiodinated β-CIT and FP-CIT on a commercially available radiochemistry synthesizer platform, performing the evaluation experiments with sodium [^131^I]iodide ([^131^I]NaI). In addition, we chose acidified H_2_O_2_ as a volatile oxidizing agent to prevent oxidizer impurities during the SPE purification.

## 2. Results

### 2.1. Manual Labeling

**Figure 1:**
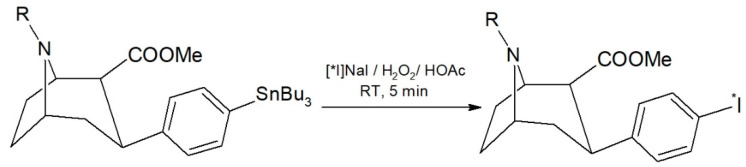
Scheme of the radioiodination of β-CIT (R = CH_3_) using acidified H_2_O_2_ as an oxidizing agent. The reaction time was 5 min at room temperature (RT, 18–25 °C).

A radioiodo-destannylation reaction is usually manually performed, preferably carried out in a small reaction volume [11,13,14]. To evaluate the optimum reaction conditions for automation, we tested the manual radiolabeling of [^131^I]β-CIT with H_2_O_2_/HOAc as an oxidizing agent in preliminary experiments. The reaction scheme is shown in Figure 1. In two experiments with a [^131^I]NaI starting activity of 19.9–23.3 MBq, we found a near quantitative radiochemical yield of 98.0 ± 0.5% by HPLC. Moreover, the reaction mixture contained only 2.0 ± 0.5% of free iodine-131.

### 2.2. Automated Labeling and Reaction Conditions

**Figure 2:**
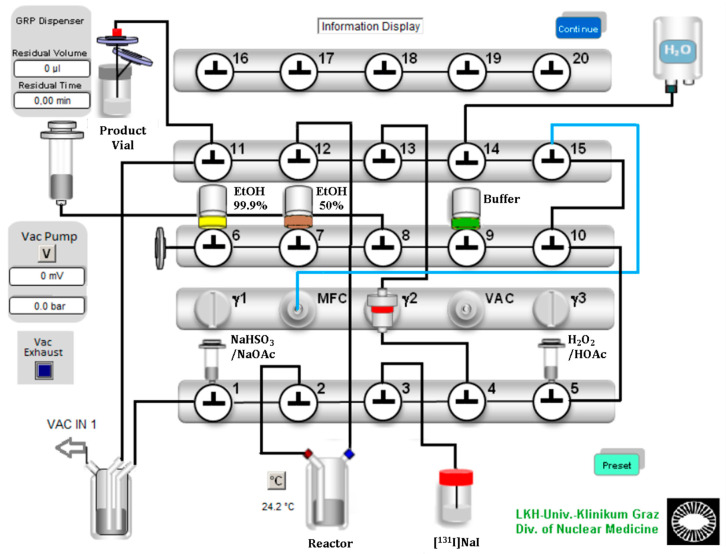
Configuration of the Scintomics GRP module for the labeling of [^131^I]β-CIT and [^131^I]FP-CIT.

Encouraged from the manual evaluation experiments, we successfully developed a suitable automated labeling route for radioiodinated β-CIT and FP-CIT on a commercially available radiochemistry synthesizer platform, using [^131^I]NaI with a starting activity of 12.2–97.3 MBq. A Scintomics GRP synthesis module was equipped with modified single-use hardware kits intended for gallium-68 peptide labeling. The configuration is shown in Figure 2. The process sequences were programmed with the Scintomics developer software, SCC.

In the configuration design, we planned a short path for the essential reagents to the reaction vessel. Furthermore, we ensured a minimum volume of over 0.5 mL for each reagent to avoid transfer losses. Valve bench one included two 3 mL syringes and the connection to the reaction vessel, which contained the ethanoic precursor solution. The first 3 mL syringe above valve 1 was filled with sodium bisulfite (NaHSO_3_)/sodium acetate (NaOAc) quenching buffer solution. The second syringe above valve 5 was filled with the oxidizing agent H_2_O_2_ (30%), which was acidified with glacial acetic acid (HOAc) and then filled up with water to a final volume of 0.5 mL. A V-vial containing the diluted [^131^I]NaI solution was coupled to the apparatus with a long syringe needle and a tube to valve 3. The second valve bench included three septum vials: 99.9% ethanol for SPE preconditioning, 50% ethanol for the elution of the product and the formulation buffer (PBS or acetate buffered saline). The third valve bench included the gas and water supply and the connection to the sterile filter and the final vial.

The process started with preconditioning of the C-18 Sep-Pak cartridge with 99.9% ethanol and water. Then [^131^I]NaI was transferred from a V-Vial to the reaction vessel by creating negative pressure with the motor syringe over valve 12. This transfer was near quantitative. Less than 2% of the radioactivity remained in the V-vial. Then, the H_2_O_2_/HOAc solution was added to the reaction vessel using the same technique.

After the reaction time had expired, the reaction mixture was quenched and neutralized with the quenching buffer solution, which was also added by applying a vacuum created by the motor syringe. The reaction mixture was aspirated, diluted with water and mixed directly in the motor syringe by a gentle nitrogen flow. Next, the reaction mixture was transferred over the C-18 Sep-Pak cartridge, where the final product was trapped. After three washing steps with water, the product was eluted from the column with 2.5 mL of 50% ethanol. Finally, after adding 16 mL of the formulation buffer through the sterile filter, the final product was ready to use.

With the described configuration, we were able to synthesize [^131^I]β-CIT with a mean radiochemical yield of 79.2% and [^131^I]FP-CIT with a mean radiochemical yield of 75.4%. The synthesis sequence and the reagents were practically identical for both compounds. However, we only extended the reaction time in the sequence from 5 min for [^131^I]β-CIT to 10 min for [^131^I]FP-CIT. Additionally, for the radiolabeling of FP-CIT we had to increase the amount of the oxidizing agent, a 30% H_2_O_2_ solution, from 40 µL (392 µmol) to 60 µL (588 µmol). The results and reaction conditions are summarized in Table 1.

### 2.3. Final Formulation and Stability

The final solutions of [^131^I]β-CIT and [^131^I]FP-CIT were visually clear and colorless. The pH of [^131^I]β-CIT formulated in PBS was 7 and the pH of [^131^I]FP-CIT formulated in acetate buffered saline was 4.3. The pH of the formulation buffer had an impact on the filtration yield of [^131^I]FP-CIT. Using PBS (pH 7.4) as a formulation buffer, 28.2% of the radioactivity was retained on the sterile filter. Using acetate buffered saline (NaCl/NaOAc pH 4.7) as a formulation buffer, the radioactivity on the filter was decreased to 2.7%. The filtration yield of [^131^I]β -CIT was not affected by the pH of the formulation buffer. The stability of [^131^I]β-CIT and [^131^I]FP-CIT was determined by thin-layer chromatography (TLC) on silica gel using acetonitrile/water (95/5, eluent 1) as a solvent system. The stability data are presented in Table 2. Both compounds are stable in their formulation buffers PBS (pH 7.4) and NaCl/NaOAc (pH 4.7) up to 72 h post preparation (radiochemical purity ≥ 95%).

### 2.4. Quality Control

Reversed phase HPLC was performed to determine the radiochemical and chemical purity of these compounds. We developed an HPLC system using a convenient RP 18 stationary phase and a mixture of acetate buffer pH 4.7/acetonitrile as the mobile phase. To analyze β-CIT we used an isocratic eluent of buffer/acetonitrile 60/40 while for FP-CIT a mixture of buffer/acetonitrile 55/45 was optimal. For calibration, standard solutions of the cold compounds (0–100 µg/mL) were injected and measured at 254 nm. We found a limit of quantification (LOQ) of 2 µg/mL for β-CIT and 6 µg/mL for FP-CIT.

**Figure 3:**
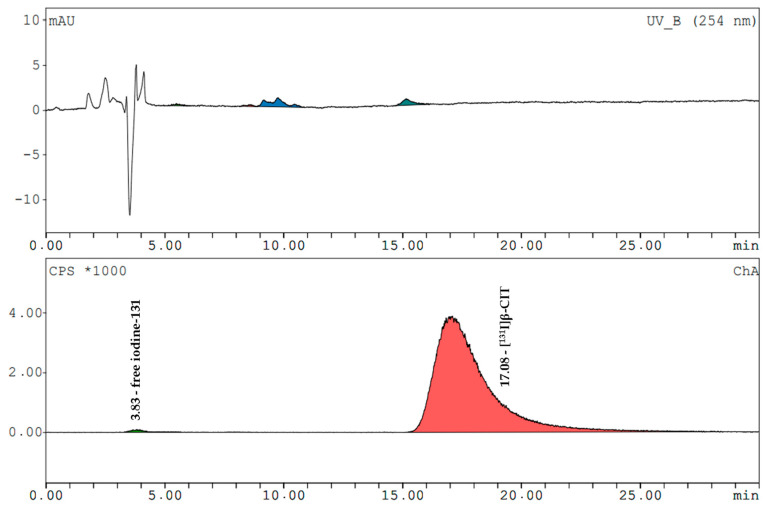
HPLC analysis of a [^131^I]β-CIT product solution. Upper trace: UV-VIS spectrum, lower trace: radiometric spectrum.

**Figure 4:**
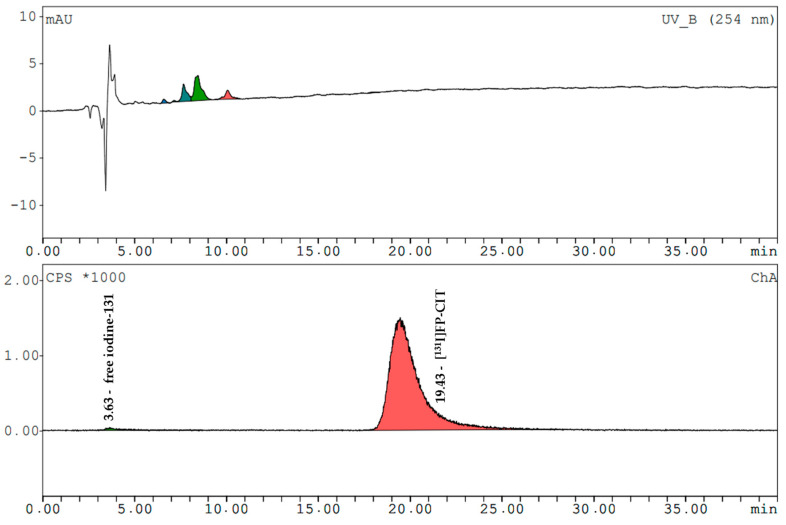
HPLC analysis of a [^131^I]FP-CIT product solution. Upper trace: UV-VIS spectrum, lower trace: radiometric spectrum.

[^131^I]β-CIT and [^131^I]FP-CIT had retention times of 17.0 ± 0.2 min and 19.3 ± 0.3 min (radiometric detection). The amount of free iodine-131 (signal at 3.7 min) in both prepared compounds never exceeded 2%. The mean radiochemical purity of [^131^I]β-CIT was 99.4% (HPLC) and 98.7% (HPLC) for [^131^I]FP-CIT. In the preparations, the content of cold compounds and the non-radioactive impurities were determined by comparing the UV trace at 254 nm with the standards. The values did not exceed the LOQ of FP-CIT. In the product solutions of [^131^I]β-CIT the average amount of non-radioactive impurities was 2.8 ± 1.9 µg/mL. Representative HPLC chromatograms (UV trace and radio-trace) of [^131^I]β-CIT and [^131^I]FP-CIT are shown in Figure 3 and Figure 4.

**Figure 5:**
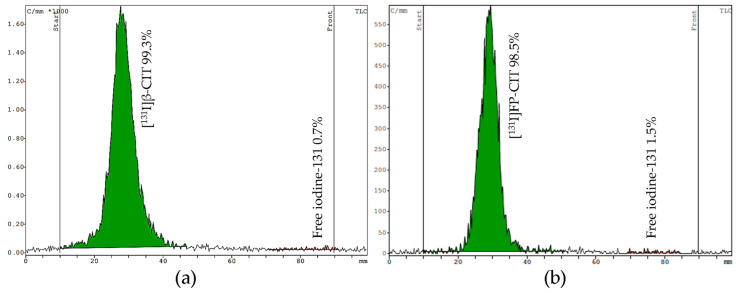
TLC radiochromatograms of [^131^I]β-CIT (**a**) and [^131^I]FP-CIT (**b**) developed in eluent II. The radiolabeled compound stays near the origin (Rf = 0.1–0.3) while free [^131^I]NaI migrates to the front (Rf = 0.9–1.0).

For TLC, we tested two mobile phases (eluent I acetonitrile/water [13] and eluent II chloroform/acidified methanol) on silica gel strips to determine free iodine and the product stability. In both systems the radiolabeled compounds stay at the origin (Rf = 0.1–0.3), while free [^131^I]NaI moves up to the front (Rf = 0.9–1.0). Both mobile phases showed good separation of the product from free iodine. However, the main advantage of eluent I is the shorter runtime of 10 min, compared to eluent II, which requires 25 min to develop the TLC plate. Representative TLC radiochromatograms of [^131^I]β-CIT and [^131^I]FP-CIT are shown in Figure 5.

## 3. Discussion

The starting point of our development was the manual labeling of [^131^I]β-CIT as a proof of concept. That procedure was described with H_2_O_2_/HCl or chloramine-T as an oxidizing agent [11,14], but our requirement was using the volatile diluted H_2_O_2_/acetic acid to prevent oxidizer impurities. In our preliminary experiments, we manually labeled [^131^I]β-CIT with 25 µg tributylstannyl precursor and a diluted [^131^I]NaI labeling solution with a starting activity of 19.9–23.3 MBq, which resulted in a near quantitative reaction.

In the further development step to an automatized synthesis, it was necessary to scale up the liquid volume of the reagents, the [^131^I]NaI labeling solution (starting activity 12.2–97.3 MBq), and the reaction mixture. The volume of each reagent should not be below 0.5 mL to avoid essential transfer losses and the precursor should directly be pipetted in the reaction vessel of the synthesizer. Therefore, the reaction volume was 2.4 mL. We showed that this reaction volume did not create poor radiochemical yields. With only 25 µg tributylstannyl precursor and with 5 min reaction time, we reached a reproducible radiochemical overall yield of nearly 80% of [^131^I]β-CIT. At the purification step [^131^I]β-CIT was easy to recover from the C-18 Sep-Pak cartridge with 2.5 mL 50% ethanol. The SPE purification effectively removed free radioactive iodine and other hydrophilic chemical impurities. Finally, the [^131^I]β-CIT was automatically sterile filtered into the product vial and diluted with 16 mL PBS buffer pH 7.4, resulting in an ethanol concentration in the product solution <10%. However, the preparation of [^131^I]FP-CIT needed more of the oxidizing agent H_2_O_2_ (60 µL instead 40 µL) and a longer reaction time (10 min instead of 5 min), for a reproducible radiochemical yield of 75%. Additionally, the choice of the formulation buffer had a significant impact on the radiochemical yield. Additionally, [^131^I]FP-CIT preparation requires a formulation buffer of pH 4.7 (NaCl/NaOAc) for a quantitative elution from the sterile filter into the product vial. [^131^I]β-CIT and [^131^I]FP-CIT were prepared in high radiochemical purity with a total synthesis time of 37 min for [^131^I]β-CIT and 42 min for [^131^I]FP-CIT.

A special feature gave the processing of up to 0.7 mL diluted radioiodine labeling solution. Nowadays, solid target technology finds its way to hospital cyclotrons. That allows local small-scale production of iodine-123 and iodine-124 for research and clinical application [15]. Moreover, our development seems to allow an easily automated radioiodination of β-CIT and FP-CIT on such sites.

## 4. Materials and Methods

### 4.1. Radioiodination and Purification

The tributylstannyl precursors TBS-CT and TBSCTFP, the non-radioactive β-CIT and FP-CIT reference standards, the labeling cassettes (SC-01), and the reagent and hardware kit (SC-01-H) were purchased from ABX (Radeberg, Germany). Additional reagents were obtained from conventional suppliers and used without further purification.

The precursors (1 mg) were diluted in 1 mL of ethanol (96%). Portions of 100 µL were dispensed into Eppendorf vials and stored at −20 °C until use. No-carrier-added [^131^I]NaI in 0.1 mL carbonate buffer was obtained from POLATOM (Otwock, Poland). V-Vials were acquired from DWK (Mainz, Germany). The Sep-Pak C18 Plus Light cartridges for solid- phase extraction were purchased from Waters (Milford, MA, USA).

For the manual radiolabeling, 25 µL of the ethanoic TBS-CT solution was pipetted into a 1.5 mL Eppendorf vial. A diluted [^131^I]NaI solution (0.2 mL, 19.9–23.3 MBq) was transferred to the vial with a 1 mL syringe. The solution was acidified with 50 µL of glacial acetic acid. Subsequently, 40 µL of 30% H_2_O_2_ were added. After a reaction time of 5 min the reaction was quenched with 100 µL of a NaHSO_3_ solution (10 mg/mL) and neutralized with 1000 µL of NaOAc (1.6 M).

For the automated radiolabeling the preassembled labeling cassettes were modified. A detailed assembly list is shown in Table 3. The ethanoic precursor solution (25 µL) was pipetted into the reaction vessel. An aliquot of the diluted [^131^I]NaI solution (12.2–97.3 MBq in 0.1 mL carbonate buffer) was pipetted into the V-Vial and diluted with 600 µL of ultrapure water. The oxidizing agent was a 30% H_2_O_2_ solution (40 µL for β-CIT and 60 µL for FP-CIT) acidified with 50 µL of glacial acetic acid and diluted with water to a final volume of 500 µL in a 3 mL syringe. The quenching buffer was prepared in another syringe by mixing 100 µL NaHSO_3_ solution (10 mg/mL) and 1000 µL of NaOAc (1.6 M). The Sep-Pak C18 cartridge was preconditioned with 5 mL of ethanol 99.9% and 5 mL of water, excluding a drying step. The reaction mixture was transferred over the cartridge, with a flowrate of 10 mL/min. Three washing steps with water (19 mL) were carried out, following a drying step in the end. The labeled compounds were eluted from the cartridge with 2.5 mL of 50% ethanol with a flowrate of 10 mL/min and transferred via a sterile filter into the final vial. Then the compound solution was diluted with 16 mL of the formulation buffer by washing the sterile filter (flowrate: 30 mL/min) and subsequent flushing with N_2_.

### 4.2. Sterile Filtration of the Product Solution

A Cathivex^®^-GV 0.22 µm filter, as well as a formulation buffer (PBS, pH 7.4), were included in the SC-01-H kit. A second formulation buffer was prepared in the laboratory, consisting of a 1:10 mixture of 0.5 M NaOAc buffer (pH 4.7) and 0.15 M NaCl solution.

### 4.3. Radiochemical Purity Testing by HPLC

HPLC was performed on an Agilent 1260 series (Waldbronn, Germany) equipped with a DAD UV detector (UV-VIS at λ = 254 nm) and a GABI Star radiometric detector (Raytest, Straubenhardt, Germany). The stationary phase was a Nucleosil 100-5 C18 290 ×3 mm column (Machery Nagel, Düren, Germany). The mobile phase consisted of a NaOAc buffer 0.1 M pH: 4.7 (A) and acetonitrile (B). Standards and radiolabeled compounds were investigated by isocratic elution (0.5 mL/min) with the following solvent compositions: A/B 60/40 for β-CIT (run time: 30 min) and A/B 55/45 for FP-CIT (run time: 40 min).

### 4.4. Radiochemical Purity Testing by TLC

TLC was performed by using Silica gel 60 plastic sheets (Merck, Darmstadt, Germany) with two different solvent systems: eluent I: acetonitrile/water 95/5 [13], eluent II: chloroform/methanol 60/40 + 0.1% HOAc. Radio TLC analysis was performed with a miniGITA Dual Head radio TLC scanner (Elysia-Raytest, Straubenhardt, Germany).

## 5. Conclusions

A reliable process for the fully automated synthesis of [^131^I]β-CIT and [^131^I]FP-CIT has been created for a Scintomics GRP synthesizer with disposable single-use cassettes. The purification of the compounds via SPE was successful and the subsequent gentle elution of the product only requires a minimal amount of ethanol. Both compounds were prepared with a radiochemical yield > 75% and a high chemical and radiochemical purity. This method demonstrates an interesting new approach for the automated radioiodination of β-CIT and FP-CIT at a high quality, either with iodine-123 or with iodine-124.

## Figures and Tables

**Table 1:** Summary of the reaction conditions and results of the automated radiolabeling of [^131^I]β-CIT and [^131^I]FP-CIT.

	[^131^I]FP-CIT (*n* = 3)	[^131^I]β-CIT (*n* = 3)
**Reaction conditions**		
Precursor [nmol]	42.1	45.6
Glacial acetic acid [µmol]	872.5	872.5
H_2_O_2_ 30% [µmol]	587.4	391.6
Reaction volume [mL]	2.4	2.4
Reaction time [min]	10	5
SPE elution reagent	50% ethanol	50% ethanol
Formulation buffer	acetate buffered saline	PBS
**Radiochemical Yield [%]**	75.4 ± 2.0	79.2 ± 0.1
**Radiochemical purity HPLC**		
Free iodine-131 [%]	1.2 ± 0.2	0.6 ± 0.2
Compound [%]	98.7 ± 0.2	99.4 ± 0.2
**Unspecific chemical impurities [µg/mL]**	≤ 6.0	2.8 ± 1.9
**Radiochemical purity TLC**		
Free iodine-131 [%]	1.4 ± 0.5	0.6 ± 0.5
Compound [%]	98.7 ± 0.7	99.4 ± 0.5

**Table 2:** Stability data of [^131^I]β-CIT (*n* = 3) and [^131^I]FP-CIT (*n* = 3).

Product Solution			Stability [%]				
Compound	Formulation Buffer	pH Value	0 h	6 h	24 h	48 h	72 h
[^131^I]β-CIT	PBS	7	99.4	98.2	99.5	99.9	97.3
[^131^I]FP-CIT	NaCl/NaOAc	4.3	98.7	98.9	98.8	98.2	96.9

**Table 3:** Assembly list for the Scintomics GRP module. H = Horizontal port, V = Vertical port.

Position	Materials/Reagents	Tubing Length	Further Details
1 H	Connection to the waste bottle tube	15.5 cm	Original part of the cassette
1 V	Quenching buffer solution (NaHSO_3_/NaOAc)		3 mL syringe
2 V	Tubing to reaction vessel (main port)	9 cm	Original part of the cassette
3 V	Tubing to the V-Vial, inlet of [^131^I]NaI	20 cm	5 mL V-Vial
4 V	Tubing to Sep-Pak C18 cartridge (inlet)	8 cm	
5 V	Acidified reducing agent (H_2_O_2_/HOAc)		3 mL syringe
5 H	Tubing to valve bench 2, valve 10 H	20 cm	Original part of the cassette
6 H	Closed		Sterifix 0.2 µm filter
6 V	Ethanol 99.9%		10 mL Vial, original reagent
7 V	Ethanol 50%		10 mL Vial, original reagent
8 V	Syringe pump	40 cm	20 mL Syringe, original part of the cassette
9 V	Formulation buffer		20 mL vial
10 H	Tubing to valve bench 1, valve 5 H	20 cm	Original part of the cassette
10 V	Tubing to valve bench 3, valve 15 H	10 cm	Original part of the cassette
11 H	Connection to the waste bottle tube	15.5 cm	Original part of the cassette
11 V	Tubing to the final vial	40 cm	20 mL vial
12 V	Tubing to reaction vessel (ventilation port)	40 cm	Original part of the cassette
13 V	Tubing to Sep-Pak C18 cartridge (outlet)	25 cm	
14 V	Tubing to water bag	39 cm	Original part of the cassette
15 H	Tubing to valve bench 2, valve 10 V	10 cm	Original part of the cassette
15 V	Tubing to N_2_ outlet	30 cm	Sterifix 0.2 µm filter

## Data Availability

Data is contained within the article.

## References

[1] Booth T.C., Nathan M., Waldman A.D., Quigley A.M., Shapira A.H., Buscombe J. (2015). The role of functional dopamine-transporter SPECT imaging in parkinsonian syndromes, part 2. AJNR.

[2] Kalia L.V., Lang A.E. (2015). Parkinson’s Disease. Lancet.

[3] Morbelli S., Esposito G., Arbizu J., Barthel H., Boellaard R., Bohnen N.I., Brooks D.J., Darcourt J., Dickson J.C., Douglas D. (2020). EANM practice guideline/SNMMI procedure standard for dopaminergic imaging in parkinsonian syndromes 1.0. Eur. J. Nucl. Med. Mol. Imaging.

[4] Brooks D.J. (2016). Molecular imaging of dopamine transporters. Ageing Res. Rev..

[5] Brooks D.J., Piccini P. (2006). Imaging in Parkinson’s disease: The role of monoamines in behavior. Biol. Psychiatry.

[6] Van Laere K., De Ceuninck L., Dom R., Van den Eynden J., Vanbilloen H., Cleynhens J., Dupont P., Bormans G., Verbruggen A., Mortelmans L. (2004). Dopamine transporter SPECT using fast kinetic ligands: 123I-FP-beta-CIT versus 99mTc-TRODAT-1. Eur. J. Nucl. Med. Mol. Imaging.

[7] Booij J., Tissingh G., Winogrodzka A., Boer G.J., Stoof J.C., Wolters E.C., van Royen E.A. (1997). Practical benefit of [123I]FP-CIT SPET in the demonstration of the dopaminergic deficit in Parkinson’s disease. Eur. J. Nucl. Med..

[8] Seibyl J.P., Marek K., Sheff K., Zoghbi S., Baldwin R.M., Charney D.S., van Dyck C.H., Innis R.B. (1998). Iodine- 123-β-CIT and Iodine- 123-FP-CIT SPECT Measurement of Dopamine Transporters in Healthy Subjects and Parkinson’s Patients. J. Nucl. Med..

[9] Kuikka J.T., Bergström K.A., Ahonen A., Hiltunen J., Haukka J., Länsimies E., Wang S., Neumeyer J.L. (1995). Comparison of iodine-123 labelled 2 beta-carbomethoxy-3 beta-(4-iodophenyl)tropane and 2 beta-carbomethoxy-3 beta-(4-iodophenyl)-N-(3-fluoropropyl)nortropane for imaging of the dopamine transporter in the living human brain. Eur. J. Nucl. Med..

[10] Coenen H.H., Mertens J., Mazière B. (2006). Iodinated Radiopharmaceuticals. Radioiodination Reactions for Pharmaceuticals. Compendium for Effective Synthesis Strategies.

[11] Goodman M.M., Kung M.P., Kabalka G.W., Kung H.F., Switzer R. (1994). Synthesis and characterization of radioiodinated N-(3-iodopropen-1-yl)-2 beta-carbomethoxy-3 beta-(4-chlorophenyl)tropanes: Potential dopamine reuptake site imaging agents. J. Med. Chem..

[12] Zea-Ponce Y., Baldwin R.M., Laruelle M., Wang S., Neumeyer J.L., Innis R.B. (1995). Simplified multidose preparation of iodine-123-beta-CIT: A marker for dopamine transporters. J. Nucl. Med..

[13] Durante A.C.R., Sobral D.V., Miranda A.C.C., de Almeida E.V., Fuscaldi L.L., de Barboza M.R.F.F., Malavolta L. (2019). Comparative study of two oxidizing agents, chloramine T and Iodo-Gen^®^, for the radiolabeling of β-CIT with Iodine-131: Relevance for Parkinson’s Disease. Pharmaceuticals.

[14] Angelberger P., Kvaternik H., Portner R., Hammerschmidt F., Bergmann H., Sinzinger H. (1995). Optimized preparation of the dopaminergic ligands 123I-Epideprid and 123I-β-CIT. Radioactive Isotopes in Clinical Medicine and Research.

[15] Lamparter D., Hallmann B., Hänscheid H., Boschi F., Malinconico M., Samnick S. (2018). Improved small scale production of iodine-124 for radiolabeling and clinical applications. Appl. Radiat. Isot..

